# Letter from St. Louis

**Published:** 1863-06

**Authors:** A. M. Leslie

**Affiliations:** Saint Louis


Dear Sir:

I beg to call to your attention that the Western Dental Society will hold its seventh annual meeting in the city of Jacksonville, Illinois, on Tuesday, the 7th of July, 1863.

This Society was organized for, and has as its special aim, the advancement of the practice of the profession of Dentistry in the West. It is desirous of having the active co-operation of every one who aims at this. A few days relief from practice spent in the interchange of views and modes of operating it is believed will tend materially to the improvement of all who will attend. You are, therefore, cordially invited to be present.

The correspondence had with members of the Profession indicate that there will be a full attendance at this meeting.

A. M. Leslie,

*Saint Louis, June 1st,* 1863. *Cor. Secretary.*

